# Effects of *Allium mongolicum* Regel and its extracts on the quality of fermented mutton sausages

**DOI:** 10.1002/fsn3.2657

**Published:** 2021-12-01

**Authors:** Lihua Zhao, Xueying Sun, Jing Wu, Lin Su, Fan Yang, Ye Jin, Meizhi Zhang, Changjin Ao

**Affiliations:** ^1^ College of Food Science and Engineering Inner Mongolia Agricultural University Huhhot China; ^2^ Erdos Environment Vocational College Erdos China; ^3^ Vocational and Technical College of Inner Mongolia Agricultural University Baotou China; ^4^ College of Animal Science Inner Mongolia Agricultural University Huhhot China

**Keywords:** *Allium mongolicum* Regel and its extracts, fatty acid, fermented sausage, free amino acid, volatile flavor

## Abstract

The study aimed to evaluate the effects of *Allium mongolicum* Regel (AMR) and its water‐ and fat‐soluble extracts on the quality of fermented mutton sausages. Sausages were produced with mutton and fat. Four treatments: CO, without *Allium mongolicum* Regel and its extracts, used as control; AMR with *Allium mongolicum* Regel; AWE with water‐soluble extract of *Allium mongolicum* Regel; and AFE with liposoluble extract from *Allium mongolicum* Regel, were produced and analyzed for pH, water activity (*a*_w_), free amino acids, fatty acids, and volatiles were, respectively, in fermented mutton sausages during processing (0, 2, 5, and 7 days). The results showed that the pH values of the liposoluble extract from *Allium mongolicum* Regel (AFE), respectively, are lower than that of sample CO at the end of fermentation and ripening. The *a*_w_ in all group of sausages significantly dropped to 0.88 at the end of ripening (Day 7). Adding *Allium mongolicum* Regel and its water‐soluble extract can improve the serine (SER) content of fermented mutton sausage. The contents of five essential amino acids (EAA) were added when adding *Allium mongolicum* Regel and its fat‐soluble extract. The total fatty acid (TFA) in the treatments increased during drying and ripening. The addition of *Allium mongolicum* Regel and its extract can increase the content of volatile flavor substances such as 3‐hydroxy‐2‐butanone, 3‐methylbutyraldehyde, hexanal, octanal, and nonanal at the later stage of maturity, so as to improve the flavor substances in fermented mutton sausage. Water‐soluble extract of *Allium mongolicum* Regel (AWE) and AFE treatments had more intense flavor at the end of ripening (Day 7). The flavor of fermented mutton sausage can be improved by adding *Allium mongolicum* Regel and its extracts into fermented mutton sausage.

## Graphical abstract

The study aimed to shed light on Allium mongolicum Regel's use in fermented mutton sausages, and provide useful information for the utilization in the design of fermented sausage processing.

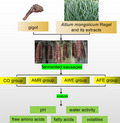

## 1 INTRODUCTION

Fermented sausage produced from meat and fat particles, salt, spices, and condiments with starter cultures fermentation and can be stored for long time due to its low water activity (0.7–0.8) and low pH (4.5–5.5) (Sun et al., 2020). The product is preferred by many consumers for their strong characteristic flavors, aroma, texture, and taste. (Ahmad & Srivastava, 2007; Wang et al., 2015). Volatile compounds belong to various classes of aldehydes, ketones, alcohols, esters, acids, terpenoids, sulfur compounds, furans, and aromatic compounds (Ciuciu et al., 2014; Marco et al., 2004; Olesen et al., 2004). It is known that the unique flavor of fermented sausage products results from the degradation of lipids, proteins and carbohydrates by tissue enzymes (protease and lipase) and microbial enzymes, the products of lipid autoxidation and the ingredients added to sausage, such as spices (Ferrocino et al., 2017; Misharina et al., 2008). The characteristics of traditional mutton products are backward processing equipment and simple methods and are usually produced in family or workshop on a small scale in China. However, with the increasing improvement of people's economic living standards, there are becoming more and more obvious that the defects of product processing methods and product characteristics. Poor equipment and low technical content are difficult to effectively control product quality and safety. For example, fermented dried mutton has a heavy smell of mutton. Spices were usually added to mutton products to cover up the odor of sheep meat. Consequently, the use of naturally occurring substances is becoming increasingly popular to improve the quality of sausages. Extracts from natural plants have unique pungent odor, which can correct the peculiar smell of food and make it to emit fragrance (Zhao et al., 2011). Thus, numerous studies have explored the potential for fermented sausages with natural plants and its extracts to improve quality defects (e.g., odor formation and production of BAs, such as diamine putrescine and cadaverine) (Marcos et al., 2013; Liu et al., 2021).

Results from such studies have shown that natural plant extracts can improve flavor and safety, physicochemical properties, as well as quality in fermented sausages (Lashgari et al., 2020). For example, Baldin et al. (2018) added microencapsulated Jabuticaba extracts showed that reduced the acceptance of color, but improved the sensory acceptance of taste. Thus, numerous studies have explored the potential for fermented sausages with natural plants and its extracts to improve quality. Some researchers using sage as antioxidant in Chinese‐style sausage showed protective effects on the deterioration of color and textural properties (Zhang et al., 2013).

*Allium mongolicum* Regel is a kind of wild vegetable with rich nutrition and unique flavor that is native to and cultivated in grasslands in northern China (Du et al.,  2019; Zhang et al., 2014). The main active ingredients of the fat‐soluble extracts of *Allium mongolicum* Regel include alkanes, flavonoids, ammonia compounds, alcohols and acids, and some phenols with aromatic odor. The main active ingredients of the water‐soluble extracts of *Allium mongolicum* Regel include cysteine derivatives, allicin, phenylpropylpyran derivatives, anthocyanin, polysaccharides, spirostene derivatives and astragaloside derivatives (Zhang, 2007). Some researchers have reported that *Allium mongolicum* Regel and its extracts could increase the contents of aromatic hydrocarbons, alcohols, ketones, aldehydes and sulfur compounds, and the content of alkanes, alkenes, esters and heterocyclic acids was reduced in muscle (Ding et al., 2021). However, to our knowledge, there is no information available regarding the effectiveness of the inclusion of *Allium mongolicum* Regel and its extracts to effect the quality in fermented mutton sausages. Consequently, the objective of the present study was to investigate the effects of addition of *Allium mongolicum* Regel and its extracts to fermented sausage on quality.

## 2 MATERIALS AND METHODS

### 2.1 Sausage sample preparation

The materials used included raw meat from the hindquarter of sheep, fat from the tail of sheep in this research obtained from market in Hohhot (Inner Mongolia, China). Starter culture was prepared by *Lactobacillus plantarum, Streptococcus pentose* and *Staphylococcus* from Meat Laboratory (College of food science and nutrition engineering, China Agricultural University, Beijing, China). *Allium mongolicum* Regel was collected in Etuokeqianqi, Ordos, Inner Mongolia. Water‐soluble extract and liposoluble extract of *Allium mongolicum* Regel were provided by Animal Nutrition Laboratory of Inner Mongolia Agricultural University (Fan et al., 2019).

All of the meat and fat were weighed in advance and cut into 4‐ to 6‐mm particle sizes. Four different sample groups of fermented mutton sausages were produced with various of *Allium mongolicum* Regel and its extracts. (1) CO: without *Allium mongolicum* Regel and its extracts; (2) AMR: with *Allium mongolicum* Regel (0.5%); (3) AWE: with water‐soluble extract of *Allium mongolicum* Regel (0.5%); (4) AFE: with liposoluble extract from *Allium mongolicum* Regel (0.5%).

The sausage was prepared according to the following formulation: lean mutton meat (90% w/w), fat (10% w/w), sucrose (0.5% w/w), salt (2.5% w/w), sodium nitrate (0.01% w/w), trite (0.007% w/w), glucose (0.5% w/w), and starter (2% w/w) (Sun et al., 2020). Mutton meat, fat, and other ingredients were mixed with 2% w/w starter cultures, which were made into 26‐mm particle size. The mixed was pickled for 12 h at 4℃ and then packed into the natural goat sausage coat and ligated with the length of 10 cm. After exhausted gas and rinsed, the sausage was suspended and fermented at 24.5℃ and 95% relative humidity (RH) for 36 h in constant temperature and humidity culture. Then separately at the condition of 14.5℃, 90% RH and 14.5℃, 80% RH for 36 and 48 h, the sausage entered drying process. Then the sausage was matured for 48 h at 14.5℃ and 70% relative humidity. Samples were obtained at 0, 2, 5, and 7 days and were taken for subsequent analysis of the experiment.

## 3 METHODS

### 3.1 Determination of pH and *a*_w_

The pH values of fermented sausages were tested using a pH meter (Model PB‐10, METTLER TOLEDO). Added 36‐ml distilled water to 4 g of each sample, stirred for 30 min and measured pH values. Water activity was determined with a moisture activity meter (Model HD‐3A, Huake Instrument Co. Ltd.).

### 3.2 Determination of free amino acid

The free amino acid content in fermented sausages was determined by automatic amino acid analyzer (L‐8900, Hitachi Limited) using the method of GB 5009.124–2016 of China (2016). Accurately weighed 30 mg of the sample dried to constant weight (accurate to 0.0001 g), added 15 ml of 6 mol/L hydrochloric acid, and hydrolyzed with the extraction temperature 90°C for 24 h. Then evaporated to dryness under reduced pressure with 1 ml of filtrate and fixed volume with 0.02 mol/L hydrochloric acid. Filtered with 0.22‐μm aperture filter, analyzed by L‐8900 automatic amino acid analyzer.

### 3.3 Determination of fatty acid

The content of fatty acid in fermented mutton sausages was profiled by gas chromatography–mass spectrometry (GC‐MS) (6890N‐5975C, Agilent Technologies Inc.) according to the literature (O'Fallon et al., 2007). Briefly, 0.5 g of minced sample were added 5 ml n‐hexane and isoacetone (3:2), added 2 ml Na_2_SO_4_ solution and centrifuged at 3000 g for 20 min. The supernatant was dried by nitrogen and then added 1 ml of anhydrous methanol, 1 ml of 12% sodium hydroxide methanol solution and 0.5 of ml n‐hexane, heated with the extraction temperature 50°C for 15 min. Cooled and added 4 ml of 10% methanol hydrochloride, heated with the extraction temperature 90°C for 2 h. Cooled and added 2 ml n‐hexane and 8 ml deionized water, mixed and remained for 10 min. Added the volume of organic layer to 10 ml and to be set aside for 5 min, adding 1 g anhydrous sodium sulfate, filtered with 0.22‐μm aperture filter, 1 µl filtrate of each sausage was injected onto GC‐MS.

In the gas chromatographic system, a HP‐88 capillary column (100 m × 0.25 mm i.d., film thickness 0.25 μm) was used. Column temperature was started from 120°C and held for 10 min and then programmed from 120 to 230°C at the rate of 3.2°C/min for 35 min. Inlet temperature was kept at 250°C. At constant voltage with 190 kpa and the detector temperature at 300℃, a sample of 1.0 μL was injected; the split ratio of the injector was 1.50.

### 3.4 Determination of volatile flavor compounds

Analysis was performed using solid‐phase microextraction (SPME) coupled to gas chromatography–mass spectrometry (GC‐MS). Two gram of sample was placed into the sample bottle, added 1 ml internal standard 2‐methyl‐3‐heptanone (1.632 μg/ml), and heated with the extraction temperature 50℃ for 30 min. The needle was desorbed in the GC injection port for 3 min at 250℃ (6890N‐5975C; Agilent Technologies) and was extended and absorbed for 40 min at 50℃ (Fonseca et al., 2013).

Agilent GC‐MS equipped with a DB‐WAX (30 m × 0.25 mm × 0.25 μm) capillary column was used for peak detection. Helium was used as the carrier gas at a flow rate of 1.2 ml/min. The injector was used in the splitless mode. The temperature was set at 40°C and maintained for 3 min, increased to 200°C at 5°C/min and maintained for 0 min, increased from 200 to 230°C at 10°C/min, and the final temperature was held for 3 min. The transfer line temperature, ion source temperature, and fourth‐stage rod were maintained at 280, 230, and 150°C. Mass spectra were obtained at 70 eV and a scan range of 40–600 amu. Volatile compounds were identified by comparison with mass spectrum data from the National Institute of Standards and Technology library database (NIST 2.0) (Jo et al., 2017).

### 3.5 Statistical analysis

Excel and SPSS 18.0 software were used for data statistics and significant difference analysis. The obtained data were analyzed by the general linear model procedure considering treatment as the main effect. Means were compared using Duncan's multiple range test, with a significance of *p* < .05.

## 4 RESULTS AND DISCUSSION

### 4.1 Effect of *Allium mongolicum* Regel and its extracts on pH and water activity (*a*_w_) of dry fermented mutton sausages

The results for pH and *a*_w_ determinations are reported during ripening time of all samples in Table 1. pH values varied from 5.18 to 5.72 during the ripening of fermented sausages. The initial pH of all groups was 5.7, and the pH value of fermented mutton sausage declined rapidly during the fermentation stage. During the process of drying and maturing, the pH value increased in all groups. Similar trends in pH were observed in study by Li et al. (2013) and Zhang et al. (2017). The pH value of sausages began to rise after 42 days of storage and stored at 20℃ was basically higher than that of sausage stored at 4℃ in our study. An increase in pH was possibly connected with protein hydrolytic activity of the starter culture, *Allium mongolicum* Regel and its extracts to form peptides, amino acids, and ammonia (Komprda et al., 2001).

**TABLE 1:** Change of pH and *a*_w_ during the ripening of the four batches of dry fermented mutton sausages

	Ripening time (days)	CO	AWE	AFE	AMR
pH	0	5.67 ± 0.04^bA^	5.72 ± 0.04^aA^	5.70 ± 0.02^aA^	5.65 ± 0.01^bA^
	2	5.22 ± 0.02^bC^	5.24 ± 0.02^aC^	5.18 ± 0.01^cC^	5.22 ± 0.02^bC^
	5	5.29 ± 0.02^aB^	5.21 ± 0.01^bC^	5.21 ± 0.01^bC^	5.27 ± 0.01^aB^
	7	5.30 ± 0.02^bB^	5.29 ± 0.01^bB^	5.38 ± 0.01^aB^	5.24 ± 0.02^cBC^
Aw	0	0.988 ± 0.001^bA^	0.991 ± 0.001^aA^	0.990 ± 0.001^abA^	0.990 ± 0.001^abA^
	2	0.979 ± 0.001^abB^	0.985 ± 0.002^aB^	0.976 ± 0.002^bB^	0.975 ± 0.002^bB^
	5	0.911 ± 0.002^aC^	0.905 ± 0.003^bAB^	0.885 ± 0.001^cC^	0.883 ± 0.001^cC^
	7	0.837 ± 0.01^aD^	0.812 ± 0.001^dC^	0.834 ± 0.01^abD^	0.826 ± 0.003^cD^

Abbreviations: CO, no addition as control; AMR, with Allium mongolicum Regel; AWE, with water‐soluble extract of Allium mongolicum Regel; AFE, with liposoluble extract from Allium mongolicum Regel; a–d means significant differences in different upper‐case letters of the same column superscript data in the table (*p* < .05); A–D means differences in different lower‐case letters of the same column superscript data in the table (*p* < .05).

The decrease in *a*_w_ values was small, and there was no obvious change at the end of fermenting (Day 2). The water activity of the sausage samples decreased steadily throughout ripening. At the end of ripening, the *a*_w_ values of all groups decreased to <0.85 that was similar to that found by Karwowska and Kononiuk (2018). The *a*_w_ value of all the experimental groups of fermented sausage samples was lower than that of the CO group (*p >* .05), which indicated that *Allium mongolicum* Regel and its extracts had impact on the *a*_w_ values of fermented mutton sausage.

### 4.2 Effect of *Allium mongolicum* Regel and its extracts on free amino acids of dry fermented mutton sausages

Free amino acids play an important role in improving the taste of sausages. The analysis of free amino acids (FAA) (expressed as mg/100 g of dry sausage samples) of dry fermented mutton sausage is reported in Table 2. It shows dry fermented with higher THR, MET, LEU, PHE, LYS, and other five essential amino acids in the AFE treatment group compared with the control group during fermentation, and the contents of total free amino acids (TFAA) in the AFE treatment group were also higher compared with the control group. The contents of arginine (ARG) were 4.821 mg/100 g and 4.74 mg/100 g in AFE group and AWE group, respectively, higher than the CO group (4.707 mg/100 g), indicating that the active components increased the content of ARG in the extract of *Allium mongolicum* Regel. The content of serine (SER) in AFE group and AWE group is higher than in CO group; the contents of glycine (GLY), methionine (MET), tyrosine (TYR), and lysine (LYS) in AFE, AWE, and AMR treatment groups were higher than those in CO group. The results showed that the active components of *Allium mongolicum* Regel and its extract were beneficial to the release of these amino acids of fermented mutton sausage. The four flavoring amino acids, glutamic acid, glycine, valine, and proline, showed a downward trend contrary to the CO group; the Maillard reaction may be the reason for the decrease or increase in these amino acids (Cordoba et al., 1994).

**TABLE 2:** Concentrations (mg/100 g) of free amino acid during the ripening of the four batches of dry fermented mutton sausages

Free amino acid	Ripening time (days)															
	0				2				5				7			
	CO	AWE	AFE	AMR	CO	AWE	AFE	AMR	CO	AWE	AFE	AMR	CO	AWE	AFE	AMR
ASP	6.261	6.714	6.691	6.895	6.777	6.458	6.630	6.718	6.985	6.884	6.761	6.827	6.709	6.694	6.803	6.678
THR	3.021	3.321	3.314	3.413	3.348	3.184	3.286	3.375	3.532	3.530	3.398	3.313	3.365	3.418	3.378	3.404
SER	2.403	2.716	2.732	2.782	2.686	2.588	2.677	2.778	2.950	2.959	2.813	2.590	2.784	2.903	2.773	2.857
GLU	11.052	12.048	11.894	12.295	12.134	11.592	11.889	12.076	12.299	12.345	12.063	11.566	11.938	11.969	11.418	11.625
GLY	3.097	3.439	3.440	3.404	3.438	3.493	3.299	3.264	3.502	3.254	3.417	3.418	3.249	3.283	3.335	3.288
ALA	4.115	4.474	4.469	4.573	4.585	4.432	4.446	4.489	4.642	4.594	4.619	4.652	4.455	4.426	4.556	4.406
CYS	1.095	1.204	1.136	1.180	1.475	0.957	1.068	1.040	0.840	1.136	1.034	1.154	1.098	1.050	1.131	1.140
VAL	3.928	4.171	4.419	4.219	4.223	4.084	4.160	4.085	4.206	4.089	4.176	4.280	4.104	3.973	4.002	3.893
MET	1.981	2.122	2.100	2.146	2.319	2.065	2.132	2.131	2.188	2.129	1.999	2.134	1.881	1.943	2.019	1.943
ILE	3.328	3.508	3.476	3.623	3.638	3.392	3.472	3.537	3.626	3.579	3.526	3.649	3.473	3.387	3.417	3.286
LEU	5.64	6.049	5.99	6.257	6.21	5.796	5.955	6.138	6.387	6.364	6.12	6.264	6.047	6.072	6.207	6.011
TYR	1.852	2.056	2.001	2.145	2.305	1.817	1.915	2.007	2.143	2.191	2.022	2.127	1.996	2.029	2.115	2.015
PHE	2.8	3.003	3	3.084	3.058	2.913	2.995	3.031	3.144	3.084	3.036	3.108	2.997	3.002	3.045	2.951
LYS	6.004	6.521	6.509	6.717	6.779	6.435	6.59	6.692	6.944	6.931	6.762	6.927	6.654	6.66	6.845	6.738
HIS	2.097	2.24	2.258	2.275	2.321	2.183	2.249	2.245	2.377	2.301	2.319	2.293	2.279	2.264	2.313	2.251
ARG	4.373	4.785	4.719	4.904	4.844	4.513	4.647	4.745	4.906	4.786	4.695	4.864	4.707	4.697	4.821	4.74
PRO	2.544	2.755	2.763	2.751	2.767	2.713	2.709	2.619	2.734	2.706	2.713	2.761	2.681	2.607	2.629	2.59
EAA	26.702	28.695	28.808	29.459	29.575	27.869	28.59	28.989	30.027	29.706	29.017	29.675	28.521	28.455	28.913	28.226
NEAA	38.889	42.431	42.103	43.204	43.332	40.746	41.529	41.981	43.378	43.156	42.456	42.252	41.896	41.922	41.894	41.59
TFAA	65.591	71.126	70.641	72.643	72.907	68.615	70.119	70.79	73.405	72.862	71.473	71.927	70.417	70.377	70.807	69.816

Abbreviations: CO, no addition as control; AMR, with Allium mongolicum Regel; AWE, with water‐soluble extract of *Allium mongolicum* Regel; AFE, with liposoluble extract from *Allium mongolicum* Regel.

The degradation of protein is an important biochemical reaction in the process of fermented sausage, which is affected by the temperature and humidity conditions in the process and the pH value and moisture content of the product. During the ripening process of fermented sausage, protein is degraded to form peptides, non‐protein nitrogen and free amino acids, and decarboxylation, deamination or Maillard reaction will further occur to produce small molecular volatile compounds, which plays a great role in the formation of taste and smell of fermented sausage. Free amino acids directly affect the formation of basic flavor of dry fermented sausage. Moreover, as a precursor of volatile compounds, FAA can generate amines and organic acids through decarboxylation and deamination and then form volatile flavor substances in the end product through a series of changes. Many free amino acids have taste, among which the sweet amino acids are glycine (GLY), threonine (THR), serine (SER), proline (PRO), and alanine (ALA); The fresh amino acids are aspartic acid (ASP) and glutamic acid (GLU); Valine (VAL), leucine (Leu), isoleucine (ILE), phenylalanine (PHE), arginine (ARG), and histidine (HIS) are bitter (Casquete et al., 2011). Diaz et al. (1997) believed that the deamination of amino acids during sausage maturation was mainly due to microbial action. A series of chemical reactions such as oxidative deamination, reductive deamination, and esterification have taken place under the action of specific enzymes, or volatile substances such as aldehydes, alcohols, ketones, pyrazines, and sulfur compounds have been directly synthesized. During the fermentation process, the contents of TFAA in the AFE group were significantly higher than those in the CO group. The increase in content was mainly caused by the increase in essential amino acids (EAA). This may be because *Allium mongolicum* Regel and its extract promote the degradation of myofibrillar protein (Feng et al., 2017; Xza et al., 2020).

### 4.3 Effect of *Allium mongolicum* Regel and its extracts on fatty acids of dry fermented mutton sausages

A different trend was noted for TFA in Table 3. The total fatty acids (TFA) were 10.163, 10.082, 7.51, and 12.178 mg/g during 0 days in the CO group, AWE group, AFE group, and AMR group, respectively. The contents of TFA decreased obviously in the middle stage of drying (Day 5) in the CO, AWE, and AFE samples. At the end of ripening (Day 7), TFA increased in all samples. The content of TFA in the three treatments was higher than that in the CO group; this may be that *Allium mongolicum* Regel and its extracts can increase lipase activity, promote fat hydrolysis, and accumulate fatty acids in the ripening stage of fermented mutton sausage.

**TABLE 3:** Concentrations (mg/g) of free fatty acid during the ripening of the four batches of dry fermented mutton sausages

Free fatty acid	Ripening time (days)															
	0				2				5				7			
	CO	AWE	AFE	AMR	CO	AWE	AFE	AMR	CO	AWE	AFE	AMR	CO	AWE	AFE	AMR
c4:0	0.552	0.519	0.709	0.635	0.544	0.471	0.065	0.071	0.059	0.128	0.063	0.084	0.079	0.041	1.283	0.008
c10:0	0.031	0.032	–	0.037	0.026	0.033	0.024	0.027	0.043	0.018	0.02	0.027	0.015	0.026	0.028	0.034
c12:0	0.057	0.054	0.036	0.078	0.054	0.068	0.043	0.046	0.047	0.037	0.034	0.051	0.031	0.051	0.053	0.066
c14:0	0.499	0.498	0.341	0.659	0.479	0.56	0.411	0.444	0.154	0.325	0.316	0.476	0.29	0.468	0.477	0.591
c16:0	2.5	2.578	1.761	3.058	2.478	2.659	2.156	2.286	0.403	0.008	0.044	2.426	0.036	2.357	2.353	2.949
c18:0	1.754	2.027	1.286	2.126	1.863	1.94	1.604	1.689	0.168	1.219	1.344	1.853	1.118	1.775	1.736	2.184
c20:0	–	–	–	–	–	–	–	–	–	0.009	0.009	0.012	0.007	0.012	0.012	0.015
c21:0	0.032	0.027	–	0.042	0.029	0.026	0.025	0.03	–	0.012	0.012	0.017	0.004	0.021	0.005	0.005
c14:1	0.032	0.033	–	0.027	0.03	0.032	0.025	0.028	0.014	0.012	0.011	0.018	0.011	0.017	0.016	0.023
c16:1	0.074	0.19	0.135	0.086	0.069	0.073	0.172	0.07	0.011	0.047	0.049	0.071	0.04	0.067	0.065	0.083
c18:1t9	0.519	0.487	0.315	0.506	0.453	0.442	0.412	0.412	0.06	0.308	0.31	0.419	0.274	0.462	0.395	0.508
c18:1c9	3.6	3.882	2.578	4.369	3.504	3.829	3.306	3.429	0.329	2.303	2.401	3.467	2.039	3.264	3.196	4.145
c20:1	0.021	–	–	0.022	–	0.029	–	0.027	0.007	0.005	0.005	0.014	0.009	0.008	0.012	0.014
c22:1n9	–	–	–	–	–	–	–	–	–	0.009	0.01	0.002	–	0.002	0.002	0.003
c18:2t6	0.041	0.038	0.028	0.037	0.038	0.036	0.034	0.037	0.007	0.033	0.028	0.041	0.028	0.036	0.033	0.043
c18:2c6	0.384	0.374	0.262	0.416	0.378	0.397	0.352	0.332	0.035	0.009	0.01	0.013	0.007	0.33	0.303	0.415
c18:3n6	–	–	–	–	–	–	–	–	–	–	–	0.005	0.006	–	0.018	0.004
c18:3n3	0.037	0.037	0.028	0.045	0.031	0.039	0.031	0.036	0.008	0.025	0.027	0.044	0.025	0.034	0.038	0.052
c20:2	–	–	–	–	–	–	–	–	–	0.005	0.005	0.007	0.004	–	–	0.006
c20:3n3	0.037	0.029	0.032	0.036	0.046	0.055	0.049	0.036	–	–	–	–	0.032	0.029	0.027	0.037
c20:5n3	–	–	–	–	–	–	–	–	–	–	–	0.006	0.004	0.005	0.006	0.007
c22:6n3	–	–	–	–	–	–	–	–	–	–	–	–	0.006	0.012	0.006	0.008
SFA	5.424	5.734	4.133	6.635	5.473	5.757	4.328	4.593	0.874	1.756	1.842	4.946	1.58	4.75	5.946	5.851
MUFA	4.241	4.591	3.029	5.009	4.055	4.404	3.914	3.965	0.42	2.683	2.785	3.991	2.374	3.821	3.687	4.775
PUFA	0.498	0.477	0.349	0.534	0.493	0.525	0.465	0.441	0.05	0.072	0.069	0.115	0.112	0.445	0.431	0.577
TFFA	10.163	10.802	7.51	12.178	10.02	10.686	8.707	8.999	1.344	4.51	4.696	9.051	4.066	9.016	10.064	11.203
PUFA/SFA	0.092	0.083	0.085	0.081	0.09	0.091	0.108	0.096	0.058	0.041	0.038	0.023	0.071	0.094	0.072	0.099

Abbreviations: CO, no addition as control; AMR, with Allium mongolicum Regel; AWE, with water‐soluble extract of Allium mongolicum Regel; AFE, with liposoluble extract from *Allium mongolicum* Regel.

In relation to the saturated fatty acid (SFA) fraction, the AWE, AFE, and AMR were 4.75, 5.946, and 5.851 mg/g of extracted lipid, respectively, at Day 7 and higher than the CO (1.58 mg/g of extracted lipid), the same results for polyunsaturated fatty acid (PUFA) fraction and monounsaturated fatty acid (MUFA) fraction. This may be due to *Allium mongolicum* Regel and its extracts can promote the decomposition of SFA to a certain extent, thus increasing the content of unsaturated fatty acids in sausages and improving the quality of fermented sausages. The fatty acid composition of each group in the process of mutton fermented sausage processing is very similar, among which palmitic acid (c16:0), stearic acid (c18:0), oleic acid (c18:1), and linoleic acid (c18:2) are the main fatty acids, which contributes greatly to the accumulation of fatty acids. Adding *Allium mongolicum* Regel and its extracts can reduce the content of stearic acid (c18:0), oleic acid (c18:1), and sheep waxy acid (c10:0) and increase the content of palmitic acid (c16:0) and linoleic acid (c18:2c6), which can significantly affect the sausage flavor. In the process of maturation and drying, it is decomposed into fatty acids and low‐grade glycerides by endogenous lipase and microbial lipase. These substances can affect the flavor of sausage and can be used as substrates to further produce flavor substances (Coutron‐Gambotti & Gandemer, 1999; Hernández et al., 1998).

PUFA was found to decrease in control samples from 0.498 mg/g at day 0–0.112 mg/g at day 7 which indicated the susceptibility of PUFA to oxidation due to oxidative and hydrolytic reactions (Channon and Trout, 2002; Feng et al., 2020). It was observed that AMR samples contained a significantly higher proportion of PUFA after 7 days of refrigerated storage, which were 0.577 mg/g, respectively, compared with control (0.112 mg/g), indicating the protective effects of *Allium mongolicum* Regel to lipid oxidation during storage. It was also noted that no significant difference was observed between AWE and AFE samples.

### 4.4 Effects of *Allium mongolicum* Regel and its extracts on volatile flavor during fermentation and ripening

The volatile flavor components of fermented sausage mainly come from spices, lipid oxidation, and flavor compounds formed by tissue enzymes (proteases and lipases) and microbial enzymes to degrade lipids, proteins, and carbohydrates. The results showed that the flavor substances of fermented meat products included aldehydes, ketones, alcohols, esters, acids, terpenoids, sulfur compounds, furans, and aromatic compounds (Marco et al., 2004; Mateo & Zumalacárregui, 1996). 9, 21, 23, and 17 kinds of volatile flavor components were detected in fermented mutton sausage after enema (Day 0), fermentation (Day 2), mid‐drying (Day 5), and ripening (Day 7) (Table 4).

**TABLE 4:** Concentrations (µg/100 g) of volatile flavor during the ripening of the four batches of dry fermented mutton sausages

Time	Volatile flavor	Odor property (Venskutonis, 2010; Smit et al., 2009; Yang et al., 2020)	Ripening time (days)															
			0				2				5				7			
			CO	AWE	AFE	AMR	CO	AWE	AFE	AMR	CO	AWE	AFE	AMR	CO	AWE	AFE	AMR
7.03	Acetic acid ethenyl ester		–	–	–	–	47.91	6.79	14.5	12.68	–	–	–	–	–	–	–	–
7.17	2,3‐Butanedione	Sweet cream	–	–	–	–	42.34	58.03	97.99	75.3	–	–	–	–	–	–	–	–
8.159	Acetic acid	Sour	–	–	–	–	96.52	14.94	8.17	13.08	–	–	–	–	–	–	–	–
9.527	3‐methyl‐butanal	Nut	–	–	–	–	10.95	7.93	9.07	15.53	4.46	3.53	3.87	5.55	12.03	12.36	28.94	13.75
9.863	3‐hydroxy‐2‐butanone		–	–	–	–	1128.59	318.68	534.55	513.20	87.15	86.94	62.63	112.63	85.20	161.44	278.13	168.56
10.593	2‐Pentanol		–	–	–	–	52.57	3.22	10.41	3.90	–	–	–	–	–	–	–	–
10.666	3‐methyl‐1‐butanol		–	–	–	–	22.23	3.46	11.65	–	–	–	–	–	–	–	–	–
11.036	Dimethyl disulfide	Garlic, onion	–	–	–	–	–	–	–	–	–	1.95	4.94	2.30	–	–	–	–
12.609	2,3‐Butanediol		–	–	–	–	26.29	5.27	1.10	9.05	32.89	17.15	28.88	19.87	25.96	18.30	129.49	17.10
12.733	Hexanal	Grass, green	29.00	9.63	24.94	12.73	432.66	54.57	114.16	218.38	95.14	72.6	52.95	110.93	154.81	155.60	365.04	148.78
14.642	(E)‐2‐Hexenal	Green, apple	–	–	–	–	–	–	–	199.69	–	–	–	2.80	–	–	–	–
15.307	o‐Xylene	Sweet, flower, geranium	–	–	–	–	–	–	–	–	3.75	5.46	1.92	3.03	–	–	–	–
16.108	Styrene	Balsamic, gasoline	–	–	–	–	10.82	–	–	7.17	6.98	10.85	8.05	8.68	13.91	3.50	8.06	6.40
16.308	Heptanal	Fat, almond, green	7.01	2.71	5.84	16.31	65.64	11.41	22.40	32.27	20.75	18.39	12.93	13.69	26.57	32.27	84.21	23.56
16.997	Methyl2‐propenyl disulfide		–	–	–	–	–	–	–	–	–	–	2.82	1.74	–	–	–	–
17.601	1R‐à‐Pinene		–	–	–	–	–	–	–	–	10.87	7.11	9.99	6.46	12.45	8.50	15.79	12.89
18.601	Benzaldehyde	Cherry, nutty, almond苦杏仁味	–	–	–	–	–	–	–	–	2.28	6.16	5.48	1.48	–	–	–	–
19.01	Dimethyl trisulfide		–	–	–	–	–	–	–	–	–	–	5.29	2.19	–	–	5.32	3.92
19.173	á‐Pinene		–	–	18.3	19.17	31.62	17.91	24.62	31.63	10.69	7.74	10.78	10.62	31.07	17.91	44.47	18.86
19.421	á‐Myrcene		–	–	–	–	36.87	4.96	–	9.14	6.19	3.35	6.54	3.36	25.74	9.71	29.03	7.71
19.834	Octanal	Green, sweet, nut	1.96	2.55	1.79	19.83	16.72	3.61	5.402	5.25	5.22	4.57	3.59	6.22	10.48	11.75	21.26	8.45
20.039	à‐Phella‐rene		–	–	–	–	–	–	–	–	3.24	2.18	3.23	–	13.22	–	8.08	–
20.251	3‐Carene		1.15	7.54	36.75	20.25	56.79	48.68	46.79	66.32	42.77	31.77	54.19	23.11	103.42	68.81	155.43	43.71
20.728	m‐Cymene		4.54	4.59	12.27	20.73	16.51	15.76	18.30	19.93	10.54	17.3	19.77	13.71	32.78	16.58	35.57	12.11
20.869	D‐Limonene	Green, apple, citrus, min	4.65	4.45	54.34	20.87	58.23	60.06	50.07	70.79	45.86	38.24	64.68	24.8	187.38	79.92	212.41	74.02
22.882	3,4‐dimethyl‐styrene		–	–	–	–	3.71	1.86	5.75	4.64	–	1.25	6.02	5.88	–	–	–	–
23.158	Nonanal	Fat, citrus, green	2.60	1.88	3.62	23.16	18.11	6.86	4.88	8.36	7.79	7.79	6.90	8.52	21.16	21.76	46.17	16.15
32.745	Caryophyllene		41.54	9.56	13.96	10.61	7.04	3.57	3.94	6.36	7.44	5.91	13.94	1.26	41.54	9.56	13.96	10.61

Abbreviations: CO, no addition as control; AMR, with Allium mongolicum Regel; AWE, with water‐soluble extract of Allium mongolicum Regel; AFE, with liposoluble extract from *Allium mongolicum* Regel.

Vinyl acetate and acetic acid are unique volatile substances at the end of fermentation, which were not detected in the stage of drying and ripening. Volatile compounds 3‐hydroxy‐2‐butanone and 2,3‐butanediol from carbohydrate metabolism; heptaldehyde, octanaldehyde, nonanal, 3‐methylbutyraldehyde from protein degradation; and pinene, 3‐carene, m‐umbelliferone, D‐limonene, and caryophyllene from spices are the main volatile flavor substances of fermented mutton sausage. At the end of drying, dimethyl disulfide, methyl 2‐propylene disulfide, and dimethyl trisulfide were detected in AFE and AMR samples, without it in CO and AWE samples. It is concluded that dimethyl disulfide, methyl 2‐propylene disulfide, and dimethyl trisulfide are the main volatile flavor compounds in *Allium mongolicum* Regel and fat‐soluble samples. A large number of pinene, 3‐carene, m‐cymene, D‐limonene, and caryophyllene were detected at the end of ripening (Day 7). Terpenes in AFE samples were higher than in control group and other treatments. Adding *Allium mongolicum* Regel and its extracts can increase the content of volatile flavor substances such as 3‐hydroxy‐2‐butanone, 3‐methyl butyraldehyde, hexanal, octanal, and nonanal and also increase the volatile flavor substances such as dimethyl disulfide, methyl 2‐propylene disulfide, and dimethyl trisulfide for improving the flavor substances at the end of ripening (Day 7) in fermented mutton sausage.

## 5 CONCLUSIONS

The use of the *Allium mongolicum* Regel and its extracts resulted in a change in some of the free amino acid, fatty acid, and volatile flavor composition and an effective decrease in pH and *a*_w_. The pH fell quickly during fermentation (Day 0 to Day 2, Table 1) and together with a lower aw (0.83 at Day 7), assured the safety and improved the quality and shelf life of dry fermented mutton sausages.

It can be concluded that *Allium mongolicum* Regel and its extracts can improve the nutritional advantages of dry fermented mutton sausage with higher SFA, MUFA, PUFA, PUFA/SFA, and TFFA compared with the control at the end of ripening (Day 7). The contents of TFAA in the AFE treatment group were also higher compared with the control. Adding *Allium mongolicum* Regel and its extracts can increase the content and types of volatile flavor substances at the end of ripening (Day 7), thus improving the flavor substances in fermented mutton sausage.

## CONFLICTS OF INTEREST

All co‐authors declare that they have no conflict of interest.

## AUTHOR CONTRIBUTIONS

**Lihua Zhao:** Conceptualization (equal); Data curation (equal); Formal analysis (equal); Investigation (equal); Methodology (equal); Software (equal); Validation (equal); Visualization (equal); Writing‐original draft (equal); Writing‐review & editing (equal). **Xueying Sun:** Data curation (equal); Formal analysis (equal); Methodology (equal); Resources (equal); Software (equal); Writing‐original draft (equal); Writing‐review & editing (equal). **Jing Wu:** Formal analysis (equal); Methodology (equal); Visualization (equal). **Lin Su:** Conceptualization (equal); Methodology (equal); Resources (equal); Software (equal). **Fan Yang:** Formal analysis (equal); Methodology (equal); Resources (equal). **Ye Jin:** Conceptualization (equal); Resources (equal); Software (equal). **Meizhi Zhang:** Data curation (equal); Formal analysis (equal); Software (equal). **Changjin Ao:** Conceptualization (equal); Funding acquisition (lead); Methodology (equal); Project administration (lead); Resources (equal); Software (equal); Supervision (equal); Visualization (equal); Writing‐review & editing (equal).

## ETHICS APPROVAL

This study does not involve any human or animal testing.

## Data Availability

The data of this study are openly available.
